# Drivers of differential views of health equity in the U.S.: is the U.S. ready to make progress? Results from the 2018 National Survey of Health Attitudes

**DOI:** 10.1186/s12889-021-10179-z

**Published:** 2021-01-21

**Authors:** Vivian L. Towe, Linnea Warren May, Wenjing Huang, Laurie T. Martin, Katherine Carman, Carolyn E. Miller, Anita Chandra

**Affiliations:** 1 Patient-Centered Outcomes Research Institute (PCORI), Washington, D.C., USA; 2 RAND Corporation, 4570 Fifth Avenue, Suite 600, Pittsburgh, PA 15213 USA; 3 Robert Wood Johnson Foundation, Princeton, NJ, USA

**Keywords:** Health equity, Population health, Health disparities, Social determinants of health

## Abstract

**Objectives:**

The public health sector has long recognized the role of the social determinants of health in health disparities and the importance of achieving health equity. We now appear to be at an inflection point, as we hear increasing demands to dismantle structures that have perpetuated inequalities. Assessing prevailing mindsets about what causes health inequalities and the value of health equity is critical to addressing larger issues of inequity, including racial inequity and other dimensions. Using data from a nationally representative sample of adults in the United States, we examined the factors that Americans think drive health outcomes and their beliefs about the importance of health equity.

**Methods:**

Using data from the 2018 National Survey of Health Attitudes, we conducted factor analyses of 21 survey items and identified three factors from items relating to health drivers—traditional health influencers (THI), social determinants of health (SDoH), and sense of community health (SoC). Health equity beliefs were measured with three questions about opportunities to be healthy. Latent class analysis identified four groups with similar patterns of response. Factor mixture modeling combined factor structure and latent class analysis into one model. We conducted three logistic regressions using latent classes and demographics as predictors and the three equity beliefs as dependent variables.

**Results:**

Nearly 90% of respondents comprised one class that was characterized by high endorsement (i.e., rating the driver as having strong effect on health) of THI, but lower endorsement of SDoH and SoC. Logistic regressions showed that respondents endorsing (i.e., rated it as a top priority) all three health equity beliefs tended to be female, older, Black or Hispanic, more educated, and have lower incomes. The class of respondents that endorsed SDoH the most was more likely to endorse all three equity beliefs.

**Conclusions:**

Results suggested that people historically impacted by inequity, e.g., people of color and people with low incomes, had the most comprehensive understanding of the drivers of health and the value of equity. However, dominant beliefs about SDoH and health equity are still generally not aligned with scientific consensus and the prevailing narrative in the public health community.

## Background

In 2008 the WHO Commission on the Social Determinants of Health called reducing health inequities an “ethical imperative” [1]. In America today, racial inequity is now a significant part of the discourse, spurred by the unequal burden of the COVID-19 pandemic and nationwide demonstrations calling for police and systems reform. Incomes have been rising, but most gains have been concentrated among the top earners, resulting in increasing income inequality [2]. Inequity in the United States is driven by a lack of just and fair access to opportunity, rooted in historical and social context, including a history of racism [3]. There are emerging efforts to enact policies and even restructure government to dismantle systemic barriers that have impeded the realization of equity in the U.S. As we continue to hear demands to break down these entrenched structures, assessing prevailing mindsets about health equity becomes critical to addressing larger inequity issues.

The U.S. is currently undergoing a set of experiences that further underscore a persistent lack of equity across numerous sectors—sectors that create major disparities in health by race/ethnicity, income, education, and other inequities. Prior to the events of 2020, equity concerns had been a focus of some health departments, mayor’s offices, philanthropies, and an explicit goal of the U.S. public health community for over a decade [4]. This focus emerged from persistently poor health outcomes disproportionately affecting low-income people and people of color [5, 6], and entrenched negative influences on health in some communities that can compound over generations. Called the social determinants of health, factors such as income, education, employment, and housing (including where people live) contribute to 50% of the variability in the distribution of length and quality of life [7] and are largely responsible for the disparities in health we observe. The data from sectors that influence health are hard to ignore: Black Americans are 5.1 times more likely than White Americans to be incarcerated [8]. In 2019, Black Americans earned 61 cents for every dollar White Americans earned (and Hispanic Americans earned 74 cents), disparities which have remained largely unchanged over the last 20 years [9]. Within racial/ethnic groups, lower income people are more likely than higher income people to lack access to care and have poorer self-reported general health [5].

Despite growing scientific consensus about the social determinants’ role in achieving health equity [1, 10–14], evidence to date shows that most Americans are unaware of these health gaps, do not understand what causes them, and do not necessarily find them to be “unfair” (a cornerstone of perceptions of inequity) and thus not worthy of action. With regards to health disparities, a survey conducted in 2008–2009 based on a national sample showed that most respondents (73%) were aware of health differences between the poor and middle class people, but less than half (46%) reported awareness of health differences between White and Black Americans [15]. Furthermore, American mindsets place the responsibility for these outcomes on individual behaviors such as smoking, diet, and exercise, as well as access to clinical care as the primary drivers, and less so the social determinants of health [16–19]. For example, while 86% of respondents of the same survey considered individual behaviors to drive health, only 31% considered where a person lives to be a factor [18], a belief more commonly held by White respondents than non-White and Hispanic respondents. (A comparable survey of Wisconsin residents found similar results [19].) Finally, political and community action are often motivated by perceptions of unfairness, so an understanding of the extent to which people believe health inequity is unfair is critical to inspiring actions that improve health [20]. Previous research has examined the degree to which Americans believe health disparities and differences in access to health promoting resources are unfair, perceptions about the source of those disparities (race, income, education, etc.), and the level of support for actions to address disparities. Across studies, survey respondents perceived systemic barriers to accessing health care as being somewhat unfair yet experiencing disparate life expectancies by race/ethnicity was perceived as less unfair. In general, the belief that individual choices mainly drive health outcomes has inhibited perceptions that disparities in health outcomes are unfair and thus warranting policy action [19, 21, 22].

This evidence suggests examining whether demands for equity reported in the media truly translate into broad public support for policies to improve health equity. The lack of consensus around equity documented in the literature also calls into question the role “echo chambers” (social environments where individuals only encounter beliefs similar to their own) which can fuel polarization and perceptions of “otherness,” and have perpetuated the conditions that cause inequity and inhibited widespread public support for systems change [16–19, 22, 23]. Given that most studies on beliefs about health disparities and equity were completed 10 years ago and much has changed regarding shared dialogue, a more recent assessment of health equity views is needed [16–19].

Previous work based on a 2015–2016 national survey provided evidence of a lack of understanding about the relationship between social determinants and health equity [16]. Misalignment between beliefs in health equity as a value and the contribution of the social determinants to health outcomes highlights a potential misunderstanding about the fundamental origins of inequities in upstream drivers of health. Since the latest research on similar questions was conducted, movements like Black Lives Matter have elevated the dialogue about race inequity. This study allows us to examine whether beliefs changed as awareness increased. This research contributes further by examining perceptions of fairness around the distribution of the social determinants or health equity, which is critical for motivating community action. Past research has not examined dominant health narratives by examining the relationship between understanding of health drivers and beliefs about health equity by demographic subgroup. Finally, rather than measuring complex concepts such as social determinants with one question, this study reflects multiple aspects of complex concepts through scales and identifies groups of respondents with similar perspectives by analyzing their response patterns. To answer our research questions fully, analyses were designed to handle both adjustment and correlation across variables.

## Methods

### Study aims

This paper contributes to our understanding of the American mindset around social determinants of health and health equity with recent data from the 2018 National Survey of Health Attitudes (NSHA) [24], a survey developed and fielded by the Robert Wood Johnson Foundation (RWJF) and the RAND Corporation, to address two aims:

1. To examine what factors people in the U.S. think drive health, whether and how levels of understanding vary, and how these levels of understanding may differ by demographic characteristics.
2. To assess the relationship between someone’s level of understanding of the drivers of health and their beliefs about the importance of achieving health equity in the U.S., with attention to demographic differences.

### Survey

This study uses data from the 2018 NSHA. The NSHA was developed as part of RWJF’s efforts to understand national perspectives related to the Culture of Health, with a primary focus on the action area *making health a shared value* [24, 25]. Relevant drivers of making health a shared value include the value of health; the role that social determinants of health play in influencing health; and a shared sense of community to influence health. These drivers were operationalized through the following measures: whether respondents (1) recognized the influence of behavioral, social, physical and other factors on health, (2) had an awareness of the role of community in health, and (3) believed in the importance and fairness of health equity.

Respondents were recruited from two nationally representative online panels: the RAND American Life Panel (ALP) and the KnowledgePanel (which was administered at the time by GfK Custom Research but has since been sold to Ipsos). Both panels: (1) are nationally representative Internet panels whose members are recruited via probability-based sampling methods; (2) provide computers and Internet connections for respondents who do not have them at the time of panel recruitment; (3) compensate respondents for their participation; and (4) collect and provide demographic information about respondents [26, 27]. The implementation of the survey was identical in the two panels. We fielded the survey using the ALP because of the rich historical data collected through that panel that can be linked to new data collection [26, 27]. These historical data include not only the previous survey that we ran in the ALP in 2015, but also any other surveys previous run in the ALP. However, to boost sample size, we also conducted the survey in the KnowledgePanel. In previous work, we compared responses across the two panels [24, 25]. To test the feasibility of combining the two samples, we investigated whether there were systematic differences between responses to the two surveys, after controlling for demographic characteristics, and found no meaningful differences.

Data were collected between July 11 and August 30, 2018. The two survey efforts combined resulted in a final total sample of 7187 completed surveys: 2479 from the ALP and 4708 from the KnowledgePanel. We used data from a subset of respondents (7077) who answered the survey items on drivers of health and well-being and health equity.

### Measures

#### Beliefs about drivers of health and well-being and sense of community health

Respondents reviewed a list of 17 items, each representing a known driver of health and well-being. These items covered a range of topics, including health-related behaviors, access to health care, knowledge about health, and social and place-based factors (e.g., employment, housing quality) and were based on existing survey questions about drivers of health developed by Robert and Booske (2011) [28]. For each item, respondents were asked on a 5-point scale what effect that driver has on health and well-being, with 1 representing “no effect” and 5 representing a “very strong effect.” We dichotomized each item by grouping responses of 4 and 5 into one category representing respondents believing the driver to have a strong effect and responses of 1–3 into a category representing respondents not believing the driver to have a strong effect. An additional set of four items asked respondents about their beliefs about their community’s ability to drive health, which were adapted from the Sense of Community Index (SCI) [29, 30]. We dichotomized each item by grouping responses “mostly” and “completely” into one category representing respondents believing their community does have the ability to drive health and responses “somewhat” and “not at all” into a category representing respondents not believing their community has the ability to drive health.

#### Beliefs about health equity

A review of existing survey questions on topics related to health disparities, drivers of health, and social determinants found few existing survey questions about health equity beliefs. One previous study had surveyed U.S. residents about their views on health equity and these items were adapted for this survey by research team members with experience in health equity theory and survey development [16]. Three questions about health equity beliefs were included in order to assess performance of different types of wording. In particular, we kept the language broadly inclusive and focused on concepts of fairness, justice, and equality. To assess beliefs about health equity, respondents were asked to place a priority value to a set of three statements about opportunities to be healthy:

1. “Making sure that the disadvantaged have an equal opportunity to be healthy.”
2. “It would be unfair if some people had more of an opportunity to be healthy than other people.”
3. “Our society needs to do more to make sure that everyone has ‘an equal’/ ‘a fair and just’ opportunity to be healthy.”

Questions 1 and 2 were included in a previous survey conducted and tested by NORC [16]. Question 3 was modified from a question included in the American National Election study on equal opportunities in general, to focus specifically on opportunities to be healthy [31]. Response options for item 1 were a 3-point scale: top priority, important but not a top priority, or not a priority at all. Response options for items 2 and 3 were a 5-point scale where 1 represented “strongly disagree” and 5 represented “strongly agree.” For item 3, the item’s wording was randomized in presentation to respondents as either “an equal” or “a fair and just opportunity.” There were no differences in distribution of response patterns between the two presentations, so for analytic purposes, they were combined into one item. All three items were dichotomized by separating responses at the highest level of endorsement from the other levels for each item. Results of this study show consistency in the performance of all three items based on similar response patterns and associations with other survey items.

### Factor analyses

We assessed the 21 items representing health drivers individually and then grouped them into factors to facilitate data analysis and assessing broader linkages between drivers of health and health equity. We first conducted an exploratory factor analysis (EFA) on the items and identified three factors, which we labeled traditional health influencers (THI), social and economic determinants of health (SDoH), and sense of community health (SoC) (see Results for more information). We followed up with a confirmatory factor analysis (CFA) to examine factor loading patterns with the goal of defining groupings of items with minimum cross loadings. Model fit was evaluated for EFA and CFA to find the best fitting model for the items. Items were deleted from future analyses if they did not contribute to the differentiation of responses.

### Latent class analysis (LCA)

We used LCA to identify participant groups with similar patterns of response to the 21 items and three factors. Models specifying up to 5 classes were run and compared on their ability to tease out distinct patterns of response aligned to factors and fit statistics, including Akaike’s information criteria (AIC) [32] and Bayesian Information Criterion (BIC) [33]. These statistics assess model fit while penalizing the number of estimated parameters. Yang (2006) [34] demonstrated adjusted BIC [35] to be the best indicator of the information criteria considered for LCA. As more classes were extracted, the model fit improved until the adjusted BIC stabilized and the number of classes was still interpretable. Respondents were assigned to classes based on their highest probability of membership [33]. The classes were mutually exclusive.

### Factor mixture modeling (FMM)

FMM is a type of structural equation modeling that allows the simultaneous inclusion of factor analysis (continuous) and LCA (categorical) [36] in the estimation process [37, 38]. FMM also accommodates the estimation of covariates on both latent factors and latent classes in the same modeling step. FMM can identify profiles in the sample while simultaneously estimating a latent factor model for each profile. For simplicity, we assumed the same latent factor model for each profile (i.e., constraining all factor loading estimates to be the same across the latent classes). The inputs used for FMM were: 1) the predefined number of factors from EFA, 2) how each factor was defined by the 21 items from CFA, and 3) the number of latent classes from the best solution in LCA.

We examined how observable demographics differed across latent classes using FMM. We incorporated basic demographics such as respondent’s age, gender, race/ethnicity, marital status, employment status, education level, household size, household income, health insurance status, urbanicity, and residence in a large city. Marital status was dichotomized as married or living with a partner vs. separated, divorced, widowed, or never married. Employment status was dichotomized as working or not working. Household size comprised three categories: one, two, or three or more people living in a household. Household income was converted into a five-level categorical scale. Race consisted of the following categories: non-Hispanic White, non-Hispanic Black, Hispanic, Asian, and non-Hispanic all other races. Education level was defined as: less than high school; high school diploma; some college or Associate’s degree; and Bachelor’s degree or more.

### Logistic regression

Latent classes from FMM results were used to examine how latent class membership and observable demographic characteristics influenced respondents’ beliefs about health equity.

Latent class membership and demographics were used as predictors in three separate logistic regressions, each using one of the three belief statements as the dependent variable to identify which characteristics predict endorsement of health equity beliefs. We modeled the probability of endorsing the highest response category, i.e., the response option “top priority” for belief statement 1 and the response option “strongly agree” for belief statements 2 and 3. All analyses in this paper were carried out using Mplus V8 [39] with the exception of the logistic regressions, conducted using Stata MP 16 [40].

## Results

### Aim 1. Factors driving health

#### Identifying factors that drive health: traditional health influencers, social determinants of health, and sense of community health

Of 21 items used for EFA and CFA, we retained 18 items based on model fit and reasonable loading. The three items dropped were smoking, genetic makeup inherited from parents, and having health insurance. Three factors were extracted from these 18 items to represent health drivers: Factor 1 (F1) represents Traditional Health Influencers (THI) and is defined by four indicators; Factor 2 (F2) represents Social and Economic Determinants of Health (SDoH) and is defined by ten indicators; Factor 3 (F3) represents Sense of Community Health (SoC) and is defined by four indicators.

FMM results for factor loadings (Table 1) show how each factor is represented by its composite indicators. Loading values are all moderate to high. Among these, the highest loading indicator for F1 (THI) is *access to affordable healthcare*, for F2 (SDoH) is *housing quality,* and for F3 (SoC) is endorsement of the statement *my community works together to make positive change for health*.

**Table 1:** Factor loadings for each factor driving health, assuming fixed values across latent classes

Factors and composite indicators	Standardized loadings
**F1: Traditional Health Influencers**	
1. Access to affordable healthcare	0.66
2. Stress	0.52
3. Knowledge about health	0.63
4. Personal health practices other than smoking	0.51
**F2: Social Determinants**	
5. Having a job	0.52
6. Neighborhood options for healthy food and exercise	0.59
7. Amount of social support	0.59
8. Physical environment such as clean air or water	0.65
9. Income	0.61
10. Community safety	0.66
11. Housing quality	0.71
12. Education	0.58
13. Where a person lives	0.64
14. Race/Ethnicity	0.46
**F3: Sense of Community Health**	
15. My community can work together to improve its health	0.83
16. My community has the resources to improve its health	0.69
17. My community works together to make positive change for health	0.84
18. I know my neighbors will help me stay healthy	0.78

#### Revealing varied comprehension of drivers of health: classes of respondents by patterns of indicator and factor endorsement

**Fig. 1:**
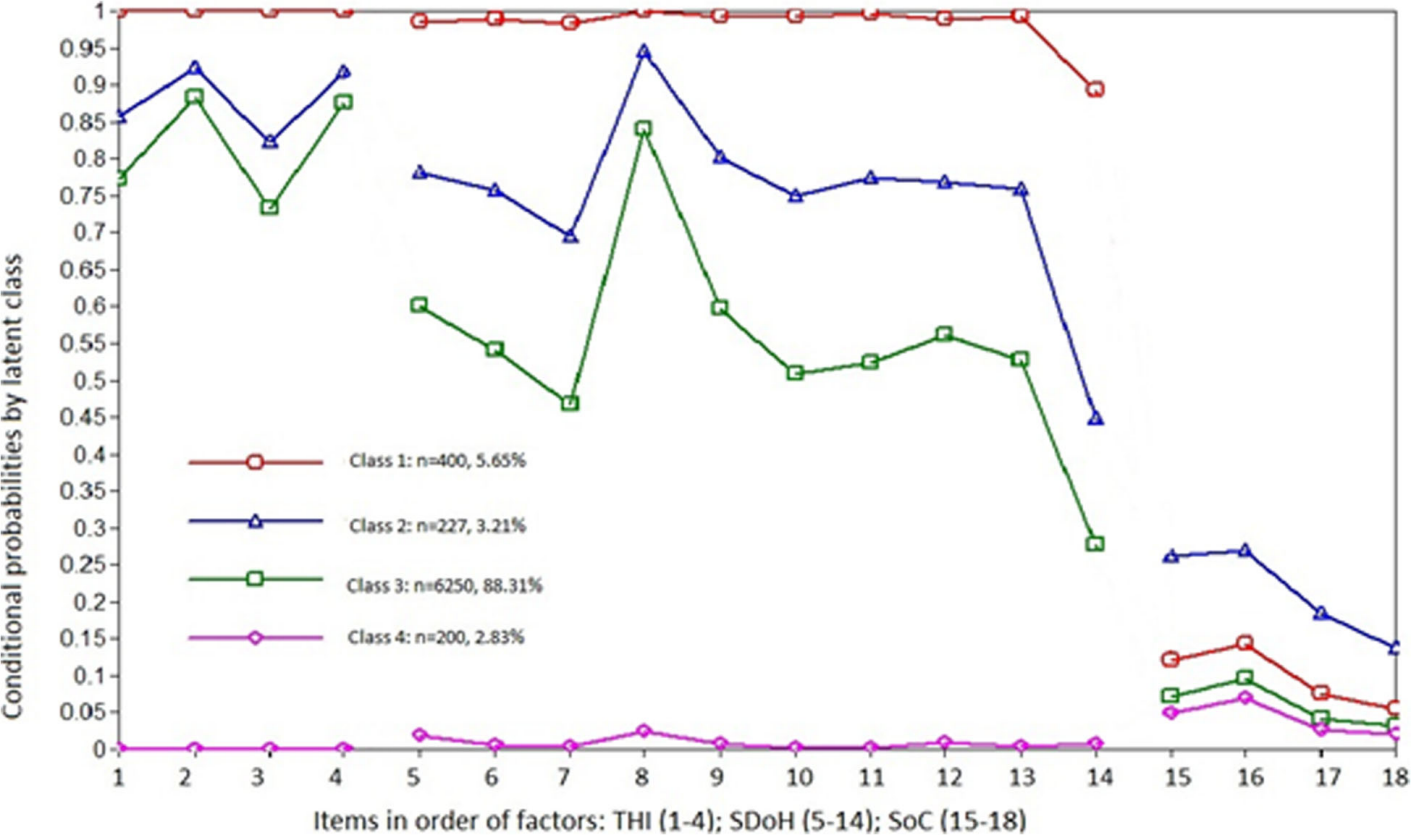
Endorsement of factors driving health by class*. *Note: Class refers to classes identified by the latent class analysis

FMM results for LCA specify “classes” of respondents based on their pattern of endorsement of health drivers. Figure 1 presents the four extracted latent classes. The x-axis presents the indicators in the following order (or see Table 1): indicators 1–4 define THI; 5–14 define SDoH; and 15–18 define SoC.

Each class is represented by a set of color-coded lines. The dots on the lines correspond to conditional probabilities (on the y-axis) of endorsing each indicator given class membership. For example, those in class 3 have approximately a 77% probability of endorsing indicator 1 (“access to affordable health care”) as being an important or very important driver of health.

Each class exhibits a distinctive pattern of indicator and factor endorsement. Class 4 is defined by a near 0 % endorsement of indicators for both THI and SDoH. Classes 1, 2, and 3 all have relatively high endorsement of indicators defining THI (greater than 70% probability for all indicators). Classes 1 and 2 are high endorsers of SDoH (greater than 70% probability for most indicators), but probability of class 3 endorsement of SDoH indicators generally hovers around 50%. Notably, for all 3 groups, indicator 14, which assesses race/ethnicity as a driver of health, was endorsed at a substantially lower percentage for classes 1, 2, and 3 compared to endorsement of other SDoH indicators. In class 2, all other SDoH indicators were endorsed at 70% of the sample or higher, but race/ethnicity was endorsed at approximately 45%. For SoC indicators, classes 1, 3, and 4 endorsed them at low percentages (all indicators under were 15%). Class 2 endorsed SoC indicators at a higher percentage (approximately 15–25%) compared to the other classes, though these percentages are still low.

Examining the distribution of survey respondents within classes, class 3 is the largest class at 88.3% of the sample. This class represents high endorsers of THI, moderate endorsers of SDoH, and low endorsers of SoC. As the majority of respondents are in class 3, it was used as the reference group in later analyses. All other classes contain very small percentages of respondents: class 1, 5.7%; class 2, 3.2%; class 4, 2.8%. Respondents of class 2 are of particular interest as they are high endorsers of both THI and SDoH, and the highest endorsers of SoC, potentially representing the group with the most comprehensive understanding of the factors that drive health.

#### Examining variation in class membership by demographics

We examined how these four classes differed by key demographics. Table 2 presents summary statistics of demographics by class.

**Table 2:** Demographic characteristics of respondents by class^a^

	Class 1	Class 2	Class 3	Class 4
*Percent of total sample*	5.7%	3.2%	88.3%	2.8%
Age mean (SD)	57.0 (15.7)	44.4 (15.1)	54.2 (15.8)	43.3 (16.7)
% Male	39.3%	64.1%	46.2%	50.8%
% Married or living with partner	55.6%	55.9%	65.4%	54.3%
% Unemployed	7.9%	5.2%	4.6%	12.1%
% Some college or more	67.2%	46.3%	74.0%	39.2%
% Household size of 1 person	23.4%	22.1%	20.3%	20.1%
% Living in rural area	17.4%	25.7%	13.1%	10.6%
% Living in large city	69.2%	39.0%	54.0%	61.8%
% Has insurance	92.4%	93.4%	94.3%	85.0%
Household income in dollars				
Less than 10,000	2.5%	22.8%	3.5%	16.6%
10,000 to 24,999	13.2%	33.1%	10.3%	17.6%
25,000 to 49,999	21.3%	23.5%	21.0%	23.6%
50,000 to 74,999	18.6%	10.3%	19.0%	16.1%
75,000 to 99,999	11.4%	2.2%	13.3%	8.0%
100,000 or more	33.3%	8.1%	32.9%	18.1%
**Race**				
Black	24.2%	33.1%	7.4%	18.1%
Hispanic	19.4%	27.2%	12.2%	20.6%
Non-Hispanic White	51.3%	29.3%	74.2%	52.8%
Asian	4.1%	5.2%	3.0%	4.5%
Other	1.0%	5.2%	3.2%	4.0%

^a^Note: Class refers to classes identified by the latent class analysis

### Class 1

LCA results indicated that Class 1 respondents were, on average, high endorsers of both THI and SDoH as drivers of health (though not SoC). Compared to other classes, class 1 respondents were least likely to be male (39%) and most likely to be living in large cities (69%). They had the second highest percentage of respondents with at least some college (67%) after class 3 (74%). They had higher household incomes compared to other classes, i.e., they have the highest percentage of respondents in the highest income category ($100,000+, 33%) and the lowest percentage of respondents in the lowest income category (less than $10,000, 2.5%). Class 1 had the second highest percentage of Black respondents (24%) after class 2 and non-Hispanic whites were the majority (51%) in this class.

### Class 2

Class 2 respondents were identified in the LCA as having the broadest comprehension about what drives health, as evidenced by high endorsement of THI and SDoH, as well as a distinctly higher endorsement of SoC, compared to other classes. Class 2 respondents were more likely to be male (64%), living in rural areas (25.7%), and least likely to be living in large cities (39%). They had lower household incomes compared to other classes, i.e., they had the highest percentage of respondents in the lowest income category (less than $10,000, 22.8%) and the lowest percentage of respondents in the highest income category ($100,000+, 8%). Class 2 had the highest percentages of Black (33%) and Hispanic (27%) respondents of all classes and class 2 is the only class in which non-Hispanic whites were not the majority (29%).

### Class 3

LCA results indicated that most of the sample was represented in class 3 (88%) and they were high endorsers of THI but had lower likelihood of endorsing SDoH and SoC. Class 3 had the highest percentages of married respondents (65%) and respondents having at least some college (74%) education. About one-third of them were in the highest household income category, similar to class 1. A vast majority of respondents in this category were non-Hispanic white (74%), which is higher than all other classes.

### Class 4

Class 4 were low endorsers of all three drivers of health. They had the highest percentage among all classes of unemployed respondents at 12.1%. They had moderate to low household incomes with 58% falling into the lowest three income categories ($49,999 and lower). Non-Hispanic whites were the majority (53%) in this class.

#### Aim 2. Association between understanding of health drivers and health equity views

Examining differences in perceptions of the importance of health equity, we observed differences by demographics and class membership across equity beliefs (Table 3).

**Table 3:** Average partial effects from regressions of demographics (independent variables) on health equity beliefs (dependent variables)

Health equity beliefs (dependent variable)	1. Making sure that the disadvantaged have an equal opportunity to be healthy	2. It would be unfair if some people had more of an opportunity to be healthy than other people	3. Our society needs to do more to make sure that everyone has ‘an equal’/ ‘a fair and just’ opportunity to be healthy
N, % endorsing highest response category	3200, 44.7%	2236, 31.3%	2920, 40.8%
Class membership (ref = class 3)			
class 1	0.24*	0.20*	0.25*
class 2	0.24*	0.14*	0.24*
class 4	−0.22*	−0.23*	−0.30*
Age	0.001*	0.002*	0.002*
Education (ref = high school diploma)			
Less than high school	0.0	−0.01	0.04
Some college, associate’s degree	0.06*	0.06*	0.07*
Bachelor’s degree or more	0.11*	0.07*	0.13*
Household size (ref = 2)			
Household size 1 person	−0.05*	−0.02	− 0.02
Household size 3 or more	−0.01	− 0.00	− 0.01
Household income (ref = between $75,000 and $124,999)			
< $24,999	0.18*	0.12*	0.16*
$25,000 to $49,999	0.06*	0.08*	0.07*
$50,000 to $74,999	0.04*	0.04*	0.04*
> $125,000	− 0.03	− 0.03	− 0.03
Has insurance (ref = none)	0.01	− 0.02	− 0.001
Being male (ref = female)	− 0.14*	− 0.08*	−0.09*
Married (ref = not married)	− 0.05*	−0.02	− 0.02
Unemployed (ref = not unemployed)	0.02	0.05*	0.07*
Race/ethnicity (ref = Non-Hispanic White)			
Black	0.17 *	0.16*	0.15*
Hispanic	0.13*	0.08*	0.09*
Asian	−0.12*	−0.05	− 0.004
Other	0.04	0.08*	0.04
Living in rural area (ref = not living in rural area)	−0.07	−0.04*	− 0.09*
Living in large city (ref = not living in large city)	−0.001	0.01	0.03*

Note: *p values <.05

Table 3 provides the average partial effect of each independent variable on the probability of endorsing each equity belief. A separate logistic regression was run for each belief. For a categorical variable, the average partial effect is interpreted as the average difference in the probability of endorsing the belief item for that category compared to the reference group, where the average is over the possible values of all the other covariates. For a continuous variable, the average partial effect represents the average effect of a one unit increase in the variable on the probability (on a scale of 0 to 1) of endorsing the belief item, where the average is over all the possible values of all the other covariates, including the continuous variable. Intuitively, the average partial effects can be interpreted in a similar manner to the coefficients in a linear probability model. For example, looking at the effect of gender on belief 1, the estimate of − 0.14 for gender indicates that on average, the probability that men endorsed belief 1 is 0.14 points lower than the probability that women endorsed this item, where the average is over all the possible values of the other covariates. Men are, on average, 0.14 points less likely to think this item is important compared to women.

Across three health equity beliefs, we observed some consistent endorsement patterns. Respondents who were more likely to endorse all three beliefs tended to be female, older, Black or Hispanic (vs. non-Hispanic white), have more education (some college or Bachelor’s degree compared to those with only a high-school degree), and have lower household incomes (less than $74,999 vs. between $75,000 and $124,999). We also found that respondents who *strongly agreed* with beliefs 2 and 3 were more likely to be unemployed and living in non-rural areas.

Class membership was examined in the logistic regression. We found the same endorsement response patterns across all three health equity beliefs by class in terms of direction of association and significance. Class 2 on average, had a significantly higher probability (.24 percentage points) of endorsing belief 1 compared to class 3 (the reference group), and in previous analyses, class 2 was found to have the highest comprehension of health drivers overall, strongly endorsing THI and SDoH and endorsing SoC higher than the other classes. Class 2 had similar patterns of significantly higher probabilities of endorsement of the two other beliefs compared to class 3 (.24 and .14, respectively). Members of class 1 had, on average, a significantly higher probability (.24 percentage points) of endorsing belief 1 compared to members of class 3, and this pattern was similar for the other beliefs. Members of class 1 were also strong endorsers of both THI and SDoH in previous analyses. By contrast, class 4 had, on average, a significantly lower probability (.22% points) of endorsing belief 1 compared to class 3 and 4’s endorsement patterns and were similar for the other beliefs. They were also weak endorsers of any health driver factors in previous analyses.

## Discussion

This study contributes to the evidence around Americans’ understanding of the factors that impact health, as well as offers new insight into the extent to which they value health equity and differences in these beliefs by subgroup. Despite efforts by the public health community, equity advocates, and a growing coalition of nonprofits, governments, and foundations, our research shows that the dominant narrative about drivers of health and health equity is powerful and resistant to change: Most Americans believe that health is predominantly influenced by individual behaviors and access to health care, as opposed to structural and social factors, and health equity is not a widespread priority.

Findings from the latent class analysis show that the majority of Americans surveyed in 2018 still lack knowledge about the social determinants of health or the role of community on health outcomes. Nearly 90% of respondents were members of a class (class 3) characterized by high endorsement of THI (including access to health care, stress, and health behaviors), but lower endorsement of SDoH (including having a job, neighborhood options for healthy food and exercise, social support, housing quality, and race/ethnicity), and very low endorsement of SoC (including beliefs that the community can work together to improve its health, has the resources to improve its health, and works together to make positive changes for health). Hallmarks of belonging in this class are that members were predominantly White (74%), educated (74% reporting at least some college education), and high-income (nearly half the members reported annual household incomes in the highest two categories). Each of these percentages were highest for this class compared to the other three classes. In contrast, the class found to have the broadest comprehension about what drives health (class 2), as evidenced by high endorsement of both THI and SDoH and the highest endorsement of SoC of all classes, was also the class representing only 3.2% of respondents. Members of this class were the most racially and ethnically diverse out of all classes, with the highest percentages of Black (33%), Hispanic (27%), and Asian (5.2%) members. This class was also the poorest, with 56% of individuals reporting a household income in the lowest two categories. The stark differences between these two classes in terms of their understanding of what drives health suggest that lived experiences based on race and/or income may be instructive about how SDoH and SoC can impact health.

Findings from the logistic regression examining health equity beliefs echoed previous findings and revealed new ones [19, 21, 22]. None of the three health equity beliefs received over 50% endorsement of the highest response category. In fact, only 31% of the sample strongly agreed that it would be unfair if some people had more of an opportunity to be healthy than other people. Respondents endorsing all three health equity beliefs tended to be female, older, Black or Hispanic, have more education, and have lower incomes. The relationship between income level and perceived importance of health equity showed a dose-response pattern in which the lower a respondent’s income level, the higher the likelihood of perceiving health equity to be important (observed across all three beliefs). In terms of class membership, class 2 (which had the broadest understanding of what drives health) had significantly higher probabilities of endorsing all three health equity beliefs compared to the reference class (class 3). Class 1 shared the same pattern of health equity belief endorsement as class 2; class 1’s similarities to class 2 include racial and ethnic diversity (24% Black and 19% Hispanic representation) and high endorsement of both THI and SDoH as drivers of health. The differences between these two groups are also important to understand. Class 1 is overall richer (45% reporting in two highest income categories) and more educated (20% more members reporting some college education) than class 3. Therefore racial/ethnic minority status was the factor most strongly associated with understanding of health drivers and the perceived value of health equity, even beyond the role of income, which was also significant.

These findings also have implications for how we understand the roots of beliefs about the SDoH and health equity, and the role of demographics in those beliefs. We found evidence that those who may have experienced disadvantage as a result of their race or income may more readily connect social and economic circumstances to health-related challenges. On the flipside, those who experience relative privilege based on their sociodemographic characteristics may fall back on cultural schemas regarding the role of individual behaviors, rather than considering structural influences [41], and prior research shows they may even actively oppose efforts to level the playing field, citing “reverse discrimination” [42]. Prior to this work, there has been limited research on the role of demographics and lived experience on beliefs about factors that determine health and values of health equity.

As with any study, there are important limitations to note. The NSHA is being fielded approximately every 3 years, but this paper only reports on the 2018 survey. As such, it will be useful to look at the relationship between health mindset and understanding and health equity, if and how that evolves over time, and why. We are potentially living through an inflection point when it comes to demands for racial equity, and it will be valuable to continue to track health equity beliefs among this nationally representative sample in future iterations of the survey. The health equity belief items used in this survey have been used previously and adapted for this study; however, further use of these items would produce more evidence for their construct validity. For this study, we used all three items in our analyses to better understand their performance. The results as shown in Table 3 indicate that these items performed consistently across a range of demographic predictors, suggesting that they were all understood similarly by respondents of different backgrounds. Additionally, the items we used to capture perspectives on health equity are mostly based on broad beliefs, and we do not know yet if different groups might respond differently to more specific aspects of health equity, such as race, income, class, etc. While we built on established scales of SDoH recognition, the items we used to assess SoC, while adapted from existing scales, are comparatively new given the limited research on this topic.

## Conclusion

Increasing inequities across health, social, and economic outcomes, which have only been highlighted by the impacts of the COVID-19 pandemic on low-income communities and communities of color, and widespread social unrest related to the topic of race, underscore the urgency of influencing Americans’ beliefs in the importance of achieving health equity. Many perceive that public sentiment has been shifting over the past decade, through movements like Occupy Wall Street, Black Lives Matter, and others, toward values of equity and dismantling historically unjust systems that have perpetuated disparate health outcomes. However, this research suggests that an understanding of specific mechanisms of inequity across all sectors, including health, may have not yet penetrated the American mindset. Without a more detailed understanding of the issues driving inequality, some Americans will be unable to participate fully in public discourse about how to make policy, economic, and system-based changes needed to shift the U.S. toward equitable outcomes.

And a current understanding of true public sentiment is important because issues looked upon favorably by the public can drive policy change (e.g., federally recognized marriage equality, where evolving positive views and widespread publicity about states’ positions on the issue played roles in bringing a case to the Supreme Court [43]). Based on what we know about the factors influencing health--factors related to the places where people live, work, and play--we must make systems more fair as a critical part of achieving health equity. People need to know what truly drives health (e.g., social determinants) so that they can understand how and why the U.S. has not yet achieved health equity. To understand the role of social determinants on health is to see that the real levers for improving health equity are through systems change, as opposed to individual behaviors or health care interventions alone. Public opinion about the impact of systems in health, the perceived unfairness of injustice embedded in those systems, and consequently, the level of public support for changing systems so that they improve health, all contribute to policy makers’ motivations to change those policies [28]. Moreover, systems change demands leadership from individuals who are well-versed in the interaction across health care, criminal justice, social services, education, and neighborhood influences on health. The perceived legitimacy of this leadership is partially determined by the predominant narratives we observe and our ability to transcend the boundaries we have established between individuals of different disciplines, political parties, and stations in life. As our research shows, siloing the call for health equity in the public health and social justice sectors has proven relatively futile in moving public opinion about the SDoH and importance of health equity. If our country is to realize the true change that recent calls to action have demanded, it will be critical to heed these insights about the dominant mindset in the U.S.

## Data Availability

Data were collected by the research team and have been deposited at ICPSR. Data are located at https://www.icpsr.umich.edu/ and can be accessed by registering to access study data.

## Abbreviations

AIC: Akaike’s information criteria
ALP: American Life Panel
BIC: Bayesian Information Criterion
CFA: confirmatory factor analysis
EFA: exploratory factor analysis
F1: Factor 1
F2: Factor 2
F3: Factor 3
FMM: Factor mixture modeling
LCA: latent class analysis
NSHA: National Survey of Health Attitudes
RWJF: Robert Wood Johnson Foundation
SDoH: Social determinants of health
SoC: Sense of community health
THI: Traditional health influences
